# Understanding the transition from stress to depression: a longitudinal mediational analysis of anxiety in adults from the Metropolitan Region of Chile

**DOI:** 10.3389/fpsyg.2025.1668518

**Published:** 2025-09-26

**Authors:** Gustavo Marcos-Escobar, Marcos E. Gómez, Wenceslao Unanue

**Affiliations:** Business School, Universidad Adolfo Ibáñez, Santiago, Chile

**Keywords:** perceived stress, anxiety, depression, longitudinal mediation, cognitive model

## Abstract

Drawing on recent advances in Beck’s cognitive model—which traditionally conceptualizes anxiety and depression as correlated but does not explicitly address their temporal ordering—this study tests whether anxiety operates as a sequential mediator linking sustained stress to depressive symptoms in a non-clinical adult population. Prior longitudinal and mediation studies have examined associations among stress, anxiety, and depression, but differences in design, population, and analytical focus limit their applicability to non-clinical adult contexts. We extend this literature with data obtained from 805 adults in the Metropolitan Region of Santiago, Chile, followed across three waves at two-month intervals during the COVID-19 pandemic, although no pre-pandemic baseline was available. Accordingly, findings should be interpreted as evidence of the stress–anxiety– depression sequence within pandemic conditions. A cross-lagged panel model (CLPM) was used to estimate the temporal relations among stress, anxiety, and depression. This statistical method accounts for the stability of each construct across repeated measurements while estimating directional relationships over time. Results confirmed a significant partial mediation: perceived stress at T1 predicted higher anxiety at T2, which in turn predicted increased depressive symptoms at T3 (standardized indirect effect *β* = 0.049, 95% CI [0.016, 0.091], *p* = 0.009). To the best of our knowledge, this is the first longitudinal study conducted with a non-clinical adult sample from the Metropolitan Region of Chile that validates this mediation sequence. The findings advance Beck’s model by demonstrating anxiety’s role as a sequential mediator, contribute methodologically through the use of a three-wave CLPM to test temporal precedence, and support preventive interventions targeting early detection of subclinical anxiety to disrupt trajectories toward depression. Together, the results update the cognitive model and inform both clinical practice and public health strategies in emotionally demanding contexts.

## 1 The cognitive framework of anxiety and depression: theoretical convergences, divergences, and sequential hypotheses

This study tests a sequential mediation pathway in which perceived stress increases anxiety over time, and this heightened anxiety, in turn, predicts later depressive symptoms. Grounded in Beck’s (1963, 1967, 1976) cognitive theory of depression, which posits that the activation of dysfunctional cognitive schemas can give rise to various emotional disorders, this study addresses a conceptual gap in the classic model. While Beck’s framework describes how such schemas underpin both anxiety and depression, it does not explicitly formalize anxiety as a sequential mediator linking stress to depression. Building on recent empirical and theoretical advances (Higa-McMillan et al., 2014; Kok et al., 2016; Anyan et al., 2020; Vrshek-Schallhorn et al., 2015), we propose and test a sequential mediational hypothesis: perceived stress activates dysfunctional schemas, anxiety emerges as an early and transitory manifestation, and depression follows as the consolidated outcome when these patterns remain unregulated. This reconceptualization positions anxiety as a sequential mediator—rather than a parallel or co-occurring condition—extending the explanatory power of the cognitive framework.

## 2 The cognitive framework of anxiety and depression: theoretical convergences and divergences

According to Beck’s cognitive theory, depressive episodes stem from the activation of ingrained schemas organized around the “negative cognitive triad,” pessimistic beliefs about oneself, the world, and the future, which distort information processing and reinforce feelings of hopelessness, worthlessness, and failure (Beck and Alford, 2009). While the classic model emphasized how past experiences shape such schemas, Beck et al. (2024) extend this framework by underscoring that these maladaptive cognitive structures also generate future-oriented expectations of threat and hopelessness, which is central to our conceptualization of anxiety as a sequential mediator. Automatic negative thoughts, including overgeneralizations and catastrophizing, sustain this maladaptive cycle. Depressive symptoms are assessed here with the Patient Health Questionnaire-9 (PHQ-9), a validated measure of symptom frequency over the past 2 weeks (Kroenke et al., 2001).

Within the same architecture, anxiety is linked to threat-oriented schemas (Beck and Clark, 1997; Beck and Emery, 2005), characterized by hypervigilance, overestimation of danger, and underestimation of coping resources. In this model, anxiety is conceptualized as a subclinical emotional state arising early in the stress response. It is measured using the GAD-7 scale, which captures key symptoms of generalized anxiety over the past 2 weeks (Spitzer et al., 2006).

Table 1 summarizes the comparative dimensions of anxiety- and depression-related schemas within Beck’s cognitive framework. Whereas anxiety is primarily linked to threat-oriented schemas that emphasize anticipated danger, vulnerability, and heightened physiological arousal, depression is associated with loss- or defeat-oriented schemas that focus on failure, hopelessness, and withdrawal. The table illustrates the distinct schema profiles of anxiety and depression in Beck’s cognitive framework, highlighting differences in thematic focus, core beliefs, biases, triggers, and emotional correlates.

**Table 1:** Comparative dimensions of anxiety- and depression-related schemas in Beck’s cognitive framework.

Dimension	Anxiety (threat-oriented schemas)	Depression (loss/defeat-oriented schemas)
Thematic focus	Anticipated threat, danger, vulnerability (Beck et al., 1985a; Beck et al., 2024)	Loss, failure, hopelessness (Beck, 1976; Beck and Alford, 2009; Beck et al., 2024)
Core beliefs (examples)	“The world is dangerous; I am defenseless”	“I am a failure; nothing will ever improve”
Cognitive biases	Selective attention to threat; catastrophizing about the future (Beck et al., 1985a)	Rumination on past loss; pessimistic inferences (Nolen-Hoeksema, 2000)
Typical triggers	Situations perceived as threatening (evaluation, uncertainty, danger cues)	Situations of loss or rejection (criticism, failure, separation)
Emotional correlates	Fear, hypervigilance, physiological arousal	Sadness, hopelessness, withdrawal

The table was constructed by synthesizing and adapting key distinctions from Beck’s (1976, Beck et al., (1985a), Beck and Alford (2009), Beck et al., (2024) works and subsequent empirical extensions (Nolen-Hoeksema, 2000).

Perceived stress—defined as the appraisal that external demands exceed coping resources (Cohen et al., 1983)—can activate both depression- and anxiety-related schemas, either in parallel or sequentially, producing complex affective trajectories. In the sequential mediator framework proposed here, stress serves as the initiating condition: it activates maladaptive schemas, triggers anxiety as an early manifestation, and, if unregulated, culminates in depressive symptomatology. This theoretical model is empirically tested through a three-wave longitudinal design, evaluating the temporal and functional validity of anxiety as a sequential mediator linking perceived stress to depression.

## 3 From stress to depression: the articulating function of anxiety

Beck’s (1963, 1967, 1976) cognitive theory of depression proposes that stressful experiences can activate dysfunctional schemas (rigid, negative beliefs about the self, the world, and the future) that distort information processing and foster hopelessness, worthlessness, and failure (Beck and Alford, 2009; Beck et al., 2024). These schemas provide a common cognitive substrate for multiple emotional disorders, establishing a theoretical foundation for both direct and indirect pathways linking stress to affective outcomes.

### 3.1 Stress to anxiety

Beck et al. (1985a), Beck and Clark (1997) and Clark and Beck (2011), extended this framework to anxiety, positing that stressors appraised as threats can directly activate danger-oriented schemas. This activation manifests in cognitive processes such as threat-oriented attentional biases, overestimation of danger, underestimation of coping resources, and repetitive negative thinking (e.g., worry, rumination) (Michl et al., 2013). Empirical findings corroborate this pathway, showing that perceived stress heightens emotional reactivity and undermines regulatory capacity, thereby increasing vulnerability to anxiety (Higa-McMillan et al., 2014).

From anxiety to depression. Although Beck’s cognitive theory did not formalize anxiety as a transitional stage in the progression toward depression, it proposed that dysfunctional schemas provide a common substrate for both disorders (Beck, 1976; Beck and Alford, 2009). Building on this framework, sustained anxiety can be understood as a mechanism that, by amplifying processes such as worry and rumination, reactivates and strengthens maladaptive schemas. Over time, this recursive cycle depletes regulatory resources, narrows attentional focus toward threat cues, and diminishes engagement in rewarding activities, thereby increasing vulnerability to depression. Longitudinal studies provide partial support for this pathway, showing that trait anxiety predicts subsequent depressive symptoms in specific populations, such as trauma-exposed samples (Kok et al., 2016). However, empirical evidence remains limited in scope, particularly in non-clinical adult populations, underscoring a theoretical gap that this study aims to address.

### 3.2 Stress directly leads to depression

Consistent with Beck’s (1976) original formulation, stress may also precipitate depressive episodes without intermediary processes by directly activating schemas of loss and hopelessness. Longitudinal and epidemiological research corroborates this direct etiological role, showing that chronic or severe stress predicts depressive outcomes even after controlling for genetic predispositions and prior episodes (Kendler et al., 2007; Vrshek-Schallhorn et al., 2015).

In sum, three pathways with theoretical and/or empirical support emerge from this framework: (a) stress exerting a direct effect on depression, (b) stress eliciting anxiety, and (c) anxiety functioning as an intermediary mechanism linking stress to depression. Collectively, these pathways provide the conceptual rationale for the analytic strategy adopted in this study. Specifically, a three-wave cross-lagged panel model (CLPM) with bootstrapped confidence intervals was employed, enabling the simultaneous estimation of direct and indirect effects while accounting for temporal stability and reciprocal influences across measurement points (Hamaker et al., 2015; MacKinnon et al., 2004).

## 4 Conceptual gaps and contributions

While Beck (1976), Beck et al., (1985b) cognitive model identifies maladaptive schemas as a shared substrate for anxiety and depression, it does not specify a temporal sequence between them. Much subsequent literature has emphasized co-occurrence. For example, the tripartite model (Clark and Watson, 1991) links both syndromes via high negative affect, differing mainly in physiological hyperarousal (anxiety) or low positive affect (depression). Similarly, Brown et al. (1998) and transdiagnostic models view them as concurrent expressions of shared vulnerabilities, and epidemiological studies (Kendler et al., 2007) report strong bidirectional associations without temporal ordering. Although prior longitudinal studies have examined associations among stress, anxiety, and depression, important gaps remain. For example, Kok et al. (2016) demonstrated that trait anxiety can mediate the link between stress and depression. However, their clinical trauma-exposed sample limits the generalizability of findings to non-clinical adults. Similarly, Anyan et al. (2018) and Anyan et al. (2020) provided evidence consistent with a sequential process in adolescent populations. Nevertheless, their results cannot be directly extrapolated to adults outside clinical settings. Finally, Jacobson and Newman (2017) examined longitudinal associations between anxiety and depression in adults but did not position anxiety as a structured sequential mediator within a stress–depression mediation framework.

These limitations converge on three unresolved issues: (a) the lack of explicit operationalization of anxiety as a sequential mediator in adult non-clinical samples, (b) insufficient methodological designs to establish temporal precedence and bidirectional effects—many prior studies relied on only two measurement waves, which preclude testing temporal ordering and separating intraindividual stability from change (Maxwell and Cole, 2007; Mitchell and Maxwell, 2013)—, and (c) limited generalizability across different populations. In our framework, anxiety is operationally defined as a sequential mediator characterized by heightened threat-oriented attentional bias, overestimation of danger, underestimation of coping resources, and increased repetitive negative thinking (e.g., worry, rumination). It is measured with the Generalized Anxiety Disorder-7 (GAD-7; Spitzer et al., 2006), which assesses symptom frequency over the past two weeks, and is temporally positioned between perceived stress assessed with the Perceived Stress Scale-14 (PSS-14; Cohen et al., 1983)—and depressive symptoms—assessed with the Patient Health Questionnaire-9 (PHQ-9; Kroenke et al., 2001).

The present study addresses these gaps by explicitly testing the sequential mediation pathway from stress to depression via anxiety in a non-clinical adult population, using a three-wave cross-lagged panel model (CLPM) with bootstrapped confidence intervals. This design allows for the estimation of both direct and indirect effects while controlling for intraindividual stability and reciprocal influences over time (Hamaker et al., 2015; MacKinnon et al., 2004). By combining a temporally sensitive analytical approach with an updated cognitive framework, this work reconceptualizes anxiety as a sequential mediator and refines the explanatory scope of Beck’s model.

## 5 Proposed model and study design

The proposed model builds directly on the theoretical rationale developed in Sections 2 and 3. Specifically, it assumes that perceived stress activates maladaptive cognitive schemas and threat-oriented processes, which increase vulnerability to anxiety. Sustained anxiety, in turn, undermines regulatory resources, fosters repetitive negative thinking (e.g., worry, rumination), and facilitates the emergence of depressive symptoms. At the same time, consistent with Beck’s (1976) original cognitive formulation and subsequent longitudinal evidence (Kendler et al., 2007; Vrshek-Schallhorn et al., 2015), stress may also exert a direct effect on depression by activating schemas of loss and hopelessness, independent of the mediating role of anxiety. Preliminary analyses of our three-wave data supported this dual structure: perceived stress predicted later anxiety, anxiety predicted subsequent depressive symptoms, and a significant indirect effect from stress to depression through anxiety was observed, while a weaker but significant direct effect from stress to depression also emerged. This dual specification provides both theoretical coherence and empirical testability, offering logical clarity and theoretical consistency, as well as empirical testability (Ashkanasy, 2016).

To examine these mechanisms, a three-wave cross-lagged panel model (CLPM) with bootstrapped confidence intervals was employed, which enables the estimation of directional effects across time points while simultaneously controlling for autoregressive stability and reciprocal influences. By incorporating repeated measures of both mediator and outcome, this analytic approach ensures temporal ordering and reduces bias in testing indirect effects (Hamaker et al., 2015; Maxwell and Cole, 2007; Mitchell and Maxwell, 2013; MacKinnon et al., 2007). Accordingly, the following hypotheses were formulated:

*H1:* Perceived stress at Time 1 will predict increased anxiety at Time 2.

*H2:* Anxiety at T2 will predict increased depressive symptoms at T3.

*H3:* Perceived stress at T1 will predict increased depressive symptoms at T3.

*H4:* Anxiety at T2 will mediate the effect of stress at T1 on depression at T3.

Consistent with Beck’s cognitive framework, we hypothesized that perceived stress at T1 would predict increased anxiety at T2 (H1), that anxiety at T2 would predict higher depressive symptoms at T3 (H2), and that perceived stress at T1 would directly predict depressive symptoms at T3 (H3). Furthermore, we posited that anxiety at T2 would mediate the longitudinal association between stress at T1 and depression at T3 (H4). We did not specify whether this mediation would be complete or partial, as Beck’s model allows for both direct and indirect effects, and we aimed to let the data inform the relative magnitude of these pathways. In addition, the inclusion of the control variable ‘fear of COVID-19’ was theoretically justified, given that pandemic-related fears have been shown to affect stress, anxiety, and depression simultaneously (Ahorsu et al., 2020). Controlling for this variable ensured that the mediation pathway captured the unique contribution of stress and anxiety, rather than reflecting pandemic-specific confounding effects.

## 6 Method

### 6.1 Procedure

This longitudinal study consisted of three measurements taken at two-month intervals and was developed according to the guidelines of the American Psychological Association and the British Psychological Society. It was approved by the university’s ethics and research committee in Chile. Data collection was carried out with the support of Netquest (www.netquest.com), an international company specializing in the management of online panel data (OPD). This company operates an OPD in Chile, serving 122,374 people, and complies with the quality standards defined by ISO 26362. In addition, Netquest is a pioneer in Latin America, holding the ISO 20252 certification, which aims to ensure high standards in market, social, and opinion research. OPDs offer significant advantages over convenience samples or face-to-face surveys, allowing data to be collected more quickly, with less bias and greater cost efficiency, especially in longitudinal studies (Porter et al., 2019). To ensure data quality, Netquest was instructed to apply the methodological guidelines of Porter et al. (2019) and to implement rigorous incentive strategies. Ongoing communication was maintained between the company and the research team. Participants were invited to complete a questionnaire designed by the researchers. An informed consent form describing the study’s objectives was included in each wave of the study. During T1, participants agreed to take part in a three-stage longitudinal study, authorizing their participation in T2 and T3. Only those who completed T1 were eligible to participate in T2, and only those who completed both T1 and T2 were invited to participate in T3.

### 6.2 Sample

A representative sample was drawn from the Netquest database in Santiago, Chile, with a proportional distribution by gender and age, based on the 2017 Chilean census. This sample accurately represents the adult population of the Metropolitan Region, which constitutes 41.18% of the national total. The first wave of data collection took place in August 2021, aiming to reach at least 479 participants who completed all three measurements. Recommendations regarding SEM sample size vary. Some authors suggest a minimum of 200 participants or 10 per parameter (Weston and Gore, 2006), while others propose at least 5 per parameter (Worthington and Whittaker, 2006). Monte Carlo simulations indicate that the required sample size may range from 30 to 460 cases, depending on multiple factors. In this study, the threshold recommended by Wolf et al. (2013) was surpassed, aligning with similar studies, such as those by Burkholder and Harlow (2003).

To strengthen the validity of the study, we implemented a three-wave longitudinal design. Cross-sectional studies, although common in stress and mental health research, are limited because they cannot establish temporal precedence and are unable to disentangle reciprocal or mediational processes among variables (Cole and Maxwell, 2003). In contrast, longitudinal designs enable the examination of intraindividual change and the testing of directional relationships over time. Specifically, having three waves makes it possible to estimate indirect effects more robustly and to model reciprocal associations between stress, anxiety, and depression, which is not feasible with two-wave designs (Little, 2013). This design, therefore, provides more substantial evidence regarding the temporal dynamics of psychological distress and enhances the interpretability of mediation mechanisms.

A post-hoc power analysis was performed to verify the adequacy of the sample size. Following recommendations that discourage post-hoc power based on observed effect sizes (Gelman, 2019), we conducted an RMSEA-based sensitivity power analysis at the model-fit level (close-fit vs. not-close-fit), which does not rely on any single observed parameter. Specifically, using *α* = 0.05, df = 450, and a close-fit null of RMSEA_H0 = 0.05, achieved power was evaluated at the observed sample size (*N* = 805) with the semPower package (Moshagen and Erdfelder, 2016). The analysis indicated Power (1 – *β*) > 0.99, confirming that the sample size was adequate to detect the hypothesized effects. This calculation is reported solely as a complementary sensitivity check of sample adequacy, rather than as a conclusive inferential test. The inclusion criteria were being over 18 years of age, residing in the Metropolitan Region, being a native Spanish speaker, and agreeing to participate in the three waves by providing informed consent. Those who had recently participated in similar research or did not meet the requirements were excluded. No additional restrictions were applied. A total of 805 adults (*M* = 43.27, *SD* = 15.54; 51.55% women) completed T1. In T2, 599 individuals participated (mean age = 45.02, *SD* = 15.67; 52.09% female), and in T3, 479 individuals (mean age = 46.37, *SD* = 15.72; 53.24% female). The sociodemographic characteristics of period T1 are presented in Table 2.

**Table 2:** Sociodemographic characteristics of participants at T1 (*N* = 805).

		Total		Male		Female	
Variable		*N*	%	*N*	%	*N*	%
Age range	18–29	213	26.46	113	29.0	100	24.1
	30–44	222	27.58	111	28.5	111	26.7
	45–59	207	25.71	96	24.6	111	26.7
	Older than 59	163	20.25	70	17.9	93	22.4
Education	Incomplete School	47	5.84	16	4.10	31	7.47
	Secondary School	116	14.41	45	11.54	71	17.11
	Incomplete associate’s degrees	70	8.70	28	7.18	42	10.12
	Associate’s degrees	272	33.79	138	35.38	134	32.29
	Bachelor’s Degrees	247	30.68	138	35.38	109	26.27
	Postgraduate/Professional	53	6.58	25	6.41	28	6.75
Marital status	Single	344	42.73	170	43.59	174	41.93
	Married	237	29.44	123	31.54	114	27.47
	Civil Union Agreement	15	1.86	10	2.56	5	1.20
	Divorced	56	6.96	22	5.64	34	8.19
	Separated	44	5.47	15	3.85	29	6.99
	Widower/widow	13	1.61	3	0.77	10	2.41
	Cohabiting	95	11.80	46	11.79	49	11.81
	Other	1	0.12	1	0.26	0	0.00
Occupation	Student	68	8.45	40	10.26	28	6.75
	Housewife	65	8.07	0	0.00	65	15.66
	Working full time	335	41.61	191	48.97	144	34.70
	Working part time	90	11.18	45	11.54	45	10.84
	Unemployed, looking for work	86	10.68	49	12.56	37	8.92
	Unemployed, not looking for work	17	2.11	4	1.03	13	3.13
	Suspended work^a^	30	3.73	14	3.59	16	3.86
	Retired	89	11.06	38	9.74	51	12.29
	With a disability, I cannot work	3	0.37	2	0.51	1	0.24
	Other	22	2.73	7	1.79	15	3.61
Income	High Income	185	22.98	121	31.03	64	15.42
	Medium income	474	58.88	210	53.85	264	63.61
	Low income	146	18.14	59	15.13	87	20.96

a Under the Employment Protection Act.

*N*, number of participants; %, percentage within total by category.

To assess dropout effects, comparisons were made between participants who completed all waves and those who dropped out after T1. Attrition rates were 25.6% between T1 (*n* = 805) and T2 (*n* = 599), and 20.0% between T2 and T3 (*n* = 479), corresponding to an overall retention rate of 59.5% across the three waves. No significant differences were found in gender (χ^2^(1) = 1.34, *p* = 0.247), perceived stress (t(803) = −1.33, *p* = 0.184), anxiety (t(803) = −1.97, *p* = 0.050), depression (t(803) = −1.79, *p* = 0.073), or fear of Covid-19 (t(803) = 0.20, *p* = 0.840). However, there was an age difference (t(737.92) = 6.34, *p* < 0.001), as participants who remained in the study tended to be older. Although this difference does not undermine the internal validity of the results, it may indicate that younger participants were more likely to discontinue participation, a pattern also documented in longitudinal survey research (Fumagalli et al., 2013), potentially limiting the generalizability of the findings to younger age groups. Even so, attrition did not compromise the validity of the study. Little's (1988) test confirmed that missing data followed a pattern of missing completely at random (MCAR), χ^2^(12) = 11.99, *p* = 0.100. Therefore, the full information maximum likelihood method (FIML) was used to handle missing data, as it provides efficient and unbiased estimates under the MCAR assumption (Newman, 2014). Nonetheless, we acknowledge that MCAR tests have limited power to detect subtle patterns of non-random missingness; thus, the reported attrition rates and demographic differences should be considered when interpreting the results. In particular, although our test supported MCAR, it is more realistic to assume that missingness in longitudinal survey research may approximate “missing at random” (MAR) rather than a strict MCAR mechanism. Under both MCAR and MAR, the use of FIML remains appropriate; however, the assumption of completely random missingness should be interpreted with caution, particularly about factors such as the type of stress or educational level.

The variables analyzed exhibited acceptable skewness and kurtosis distributions, as per Kline (2016). For perceived stress, skewness was (0.17 at T1, 0.63 at T2, 0.57 at T3) and kurtosis (−0.23 at T1, 0.42 at T2, 0.18 at T3). For anxiety, skewness was (0.50 at T1, 0.58 at T2, 0.56 at T3) and kurtosis (−0.64 at T1, −0.38 at T2, −0.41 at T3). Regarding depression, skewness values were (0.81 at T1, 1.02 at T2, 0.97 at T3) and kurtosis (0.03 at T1, 0.53 at T2, 0.48 at T3).

### 6.3 Measurements

Validated instruments with robust psychometric properties were used, and conceptual equivalence was ensured by the back-translation procedure (Brislin, 1970) when necessary.

#### 6.3.1 Perceived stress

Stress was assessed using the Perceived Stress Scale (PSS-14; Cohen et al., 1983), a Spanish validated version culturally adapted for Chile (Tapia et al., 2007). More recently, the PSS has also been validated in Chilean teachers from Copiapó, demonstrating adequate reliability (*α* ≈ 0.83) and factorial validity (Jorquera-Gutiérrez, 2023). Additionally, a transcultural adaptation of the PSS-10 was conducted in Chilean Spanish (Lagos-Riveros et al., 2023), further supporting its cultural validity. The instrument comprises 14 items that assess the frequency with which individuals perceive their lives as unpredictable, uncontrollable, and stressful over the last month. Responses were recorded on a Likert-type scale from 0 (never) to 4 (very often). Examples of items include “In the past month, how often have you felt that you could not control the important things in your life?” and “In the past month, how often have you felt that difficulties were piling up so much that you could not overcome them?”

#### 6.3.2 Anxiety

Anxiety was measured with the Generalized Anxiety Disorder scale (GAD-7; Spitzer et al., 2006), using the Spanish culturally adapted version validated by García-Campayo et al. (2010). In Chile, the GAD-7 has also been validated in adolescents, confirming good psychometric properties and reliability (*α* = 0.86) (Gaete et al., 2022). The scale comprises seven items that assess anxiety symptoms over the last 2 weeks. Responses are given on a Likert scale from 0 (not at all) to 3 (almost every day). Examples of items include “Feeling nervous, anxious, or on edge” and “Worrying too much about different things.”

#### 6.3.3 Depression

Depression was measured using the Patient Health Questionnaire (PHQ-9; Kroenke et al., 2001), specifically the Spanish validated version applied in Chilean primary care by Baader et al. (2012), which includes the nine items plus an additional functional impairment question, and demonstrated good sensitivity, specificity, and reliability (*α* = 0.83–0.89). Additional Chilean studies have further supported its psychometric performance, including Saldivia et al. (2019) in primary care patients and Borghero et al. (2018) in adolescents (α = 0.86). The PHQ-9 consists of 9 items, each rated on a 4-point scale (0 = “not at all,” 1 = “several days,” 2 = “more than half the days,” 3 = “nearly every day”), with a total score range of 0–27. Examples include “Feeling depressed, down, or hopeless” and “Having little interest or pleasure in doing things.”

## 7 Results

### 7.1 Analysis plan

**Figure 1:**
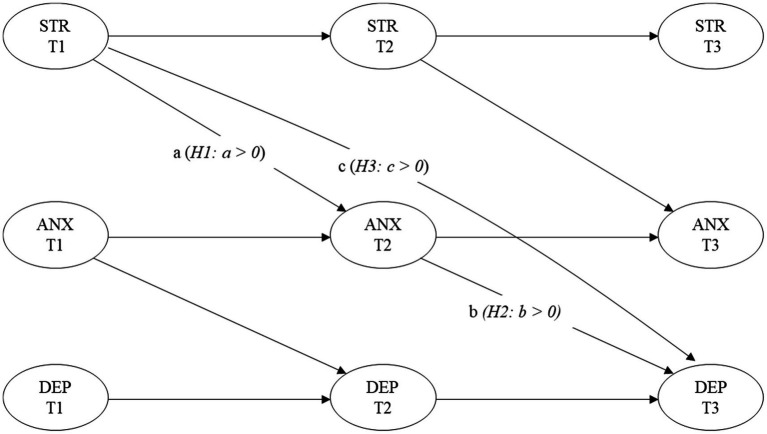
Hypothesized model. Longitudinal mediation model of anxiety, perceived stress, and depression. All variables are latent constructs. STR, perceived stress; ANX, anxiety; DEP, depression. Time 1 (T1), Time 2 (T2), and Time 3 (T3). Paths a, b, and c represent hypothesized mediation effects corresponding to H1 (a > 0), H2 (b > 0), and H3 (c > 0).

To test our hypotheses, we modeled all constructs as latent variables in order to minimize measurement errors (Finkel, 1995). To test our longitudinal mediation hypothesis, we employed structural equation modeling (SEM; Cole and Maxwell, 2003; Selig and Preacher, 2009), following the recommendations of Maxwell et al. (2011) and O'Laughlin et al. (2018). Figure 1 illustrates our hypothesized model. We implemented a panel design in which anxiety at T2 acts as a mediating variable for the association between perceived stress at T1 and depression at T3. All variables were controlled for their lagged autoregressive trajectories. Following the approach proposed by Jöreskog (1979), we incorporated correlated residuals for the observed indicators of each latent variable and allowed all latent variables to covary freely.

In our sample, the internal consistencies of the instruments were adequate. For perceived stress (PSS-14), Cronbach’s α was 0.88 at T1, T2, and T3, and the construct was modeled as a latent variable with four balanced parcels. For anxiety (GAD-7), Cronbach’s α was 0.92 at T1, 0.92 at T2, and 0.93 at T3, modeled as a latent variable using three randomly distributed parcels. For depression (PHQ-9), Cronbach’s α was 0.90 at T1, 0.91 at T2, and 0.91 at T3, modeled as a latent variable with three parcels formed by random assignment of items.

The variable fear of COVID-19 was incorporated as a control due to its well-documented associations with anxiety, depressive symptoms, and stress during the pandemic (Ahorsu et al., 2020; Alimoradi et al., 2022). Its inclusion was not intended to examine the specific impact of the pandemic per se, but rather to isolate the hypothesized stress–anxiety–depression pathway. Operationally, fear of COVID-19 was specified as an exogenous predictor at each wave, directly predicting stress, anxiety, and depression. This specification controlled for the variance attributable to the pandemic context, preventing artificial inflation of associations among the psychological variables. As a result, the longitudinal trajectories represent net relationships independent of the contextual impact of fear of COVID-19, ensuring that the observed effects reflect genuine links. Since all waves occurred during the pandemic, results should be understood within this context. Consistent with theoretical completeness criteria, we report the models that incorporate this control variable. Notably, although the model’s results and substantive conclusions remained virtually unchanged when estimated without it, the reported analyses correspond to the models with the control included. This reinforces the robustness of the findings and indicates that the fear of COVID-19 did not exert a significant influence on the tested mediation process.

Following the recommendations of Hu and Bentler (1999) and Kline (2016), model fit was evaluated using multiple indices, including the chi-square test (χ^2^), the root mean square error of approximation (RMSEA) with its 90% confidence interval, the comparative fit index (CFI), the Tucker–Lewis index (TLI), and the standardized root mean square residual (SRMR). Interpretation was guided by widely used cutoffs, with RMSEA values ≤ 0.06 (0.08), CFI/TLI ≥ 0.95 (0.90), and SRMR ≤ 0.08 considered indicative of good (acceptable) fit, while recognizing the sensitivity of χ^2^ to sample size and model complexity. Descriptive statistics are reported in Table 2, and zero-order correlations among the study variables are presented in Table 3. All structural and longitudinal mediation analyses were conducted in Mplus 8.0 (Muthén and Muthén, 2017), with indirect effects tested via bias-corrected bootstrap resampling (1,000 draws, MODEL INDIRECT). Standardized indirect effects (*β*) and 95% bias-corrected confidence intervals were reported, and statistical significance was established when the confidence intervals did not include zero.

**Table 3:** Descriptive statistics and zero-order correlations between all variables at T1, T2 and T3 (T1: *N* = 805; T2: *N* = 599; T3: *N* = 479).

	Variable	M	SD	1	2	3	4	5	6	7	8	9	10	11	12	13	14
1	Gender	1.52	0.50														
2	Age	43.27	15.54	0.05													
3	Income	4.32	1.25	0.16**	−0.22**												
4	Perceived Stress T1	24.40	9.08	0.12**	−0.29**	0.15**											
5	Perceived Stress T2	25.34	8.10	0.10*	−0.26**	0.17**	0.74**										
6	Perceived Stress T3	24.97	8.26	0.05	−0.24**	0.19**	0.74**	0.80**									
7	Anxiety T1	8.04	5.47	0.12**	−0.27**	0.17**	0.79**	0.68**	0.66**								
8	Anxiety T2	14.67	5.31	0.11**	−0.24**	0.17**	0.63**	0.79**	0.69**	0.71**							
9	Anxiety T3	14.31	5.31	0.09*	−0.19**	0.16**	0.63**	0.68**	0.78**	0.71**	0.78**						
10	Depression T1	8.10	6.37	0.09*	−0.25**	0.23**	0.71**	0.65**	0.63**	0.75**	0.61**	0.61**					
11	Depression T2	7.33	6.23	0.10*	−0.23**	0.24**	0.60**	0.74**	0.67**	0.62**	0.74**	0.66**	0.69**				
12	Depression T3	7.43	6.17	0.04	−0.22**	0.23**	0.61**	0.67**	0.76**	0.63**	0.68**	0.75**	0.72**	0.77**			
13	Covid-19 Fear T1	3.67	1.23	0.12**	0.07*	0.00	0.10**	0.01	0.09*	0.19**	0.07	0.15**	0.11**	0.00	0.03		
14	Covid-19 Fear T2	3.52	1.23	0.04	0.15**	−0.01	0.05	0.07	0.09*	0.10*	0.12**	0.13**	0.03	0.05	0.05	0.59**	
15	Covid-19 Fear T3	3.68	1.18	0.02	0.21**	−0.03	0.03	0.02	0.09	0.08	0.05	0.11*	−0.03	0.00	0.00	0.63**	0.64**

**p < 0.01; *p < 0.05.

### 7.2 Estimates

To assess the factorial validity of the scales used, confirmatory factor analyses (CFA) were performed separately at each of the three measurement waves. In all cases, a single-factor model, in which all items loaded on a single latent factor, was compared with a theoretically grounded model composed of three distinct latent factors. At time one (T1), the single-factor model showed poor fit: *χ^2^*(35) = 1053.79, *p* < 0.001, CFI = 0.864, TLI = 0.825, RMSEA = 0.190, 90% CI [0.180, 0.200], SRMR = 0.056. In contrast, the three-factor model showed a substantially better fit: *χ^2^*(32) = 161.90, *p* < 0.001; CFI = 0.983; TLI = 0.976; RMSEA = 0.071, 90% CI [0.060, 0.082]; SRMR = 0.022. The difference between models was significant, *Δχ^2^*(3) = 891.89, *p* < 0.001. This procedure was replicated at T2. The one-factor model again presented an inadequate fit: *χ^2^*(35) = 716.63, *p* < 0.001, CFI = 0.875, TLI = 0.839, RMSEA = 0.180, 90% CI [0.169, 0.192], SRMR = 0.051. The three-factor model, by contrast, showed a markedly superior fit: *χ^2^*(32) = 102.53, *p* < 0.001, CFI = 0.987, TLI = 0.982, RMSEA = 0.061, 90% CI [0.048, 0.074], SRMR = 0.017. The difference between models was significant, *Δχ^2^*(3) = 614.11, *p* < 0.001. Similarly, at T3, the one-factor model showed an unsatisfactory fit: *χ^2^*(35) = 616.38, *p* < 0.001, CFI = 0.872, TLI = 0.836, RMSEA = 0.186, 90% CI [0.173, 0.199], SRMR = 0.051. The three-factor model again presented a better fit: *χ^2^*(32) = 80.97, *p* < 0.001, CFI = 0.989, TLI = 0.985, RMSEA = 0.057, 90% CI [0.041, 0.072], SRMR = 0.019. The difference between models was statistically significant, Δχ^2^(3) = 535.41, *p* < 0.001. Taken together, these results provide robust evidence supporting the discriminant factorial validity of the three constructs analyzed, justifying their treatment as conceptually and empirically differentiated dimensions over time.

To assess metric invariance, two versions of a nine-factor latent model across waves were compared. First, a base model with no restrictions on factor loadings (the free model) was estimated. This model presented an adequate fit: *χ^2^*(369) = 1320.06, *p* < 0.001, CFI = 0.952, TLI = 0.944, RMSEA = 0.057, 90% CI [0.053, 0.060], and SRMR = 0.025. Subsequently, a second model was estimated, imposing equality restrictions on all factor loadings (the restricted model). The fit of this model was also adequate and virtually identical to the previous one: *χ^2^*(383) = 1332.09, *p* < 0.001, CFI = 0.952, TLI = 0.946, RMSEA = 0.055, 90% CI [0.052, 0.059], SRMR = 0.026. The comparison between the two models showed a non-significant chi-square difference: *Δχ^2^*(14) = 12.03, *p* = 0.604. Furthermore, no change was observed in the CFI value (ΔCFI = 0.000), indicating that the restrictions imposed did not deteriorate fit quality. According to the criteria proposed by Cheung and Rensvold (2002), these results support metric invariance, which allows us to assume that the relationships between the items and the latent constructs remain stable over time. Therefore, the restrictions were maintained in the subsequent structural analyses.

### 7.3 Structural models and comparisons

**Figure 2:**
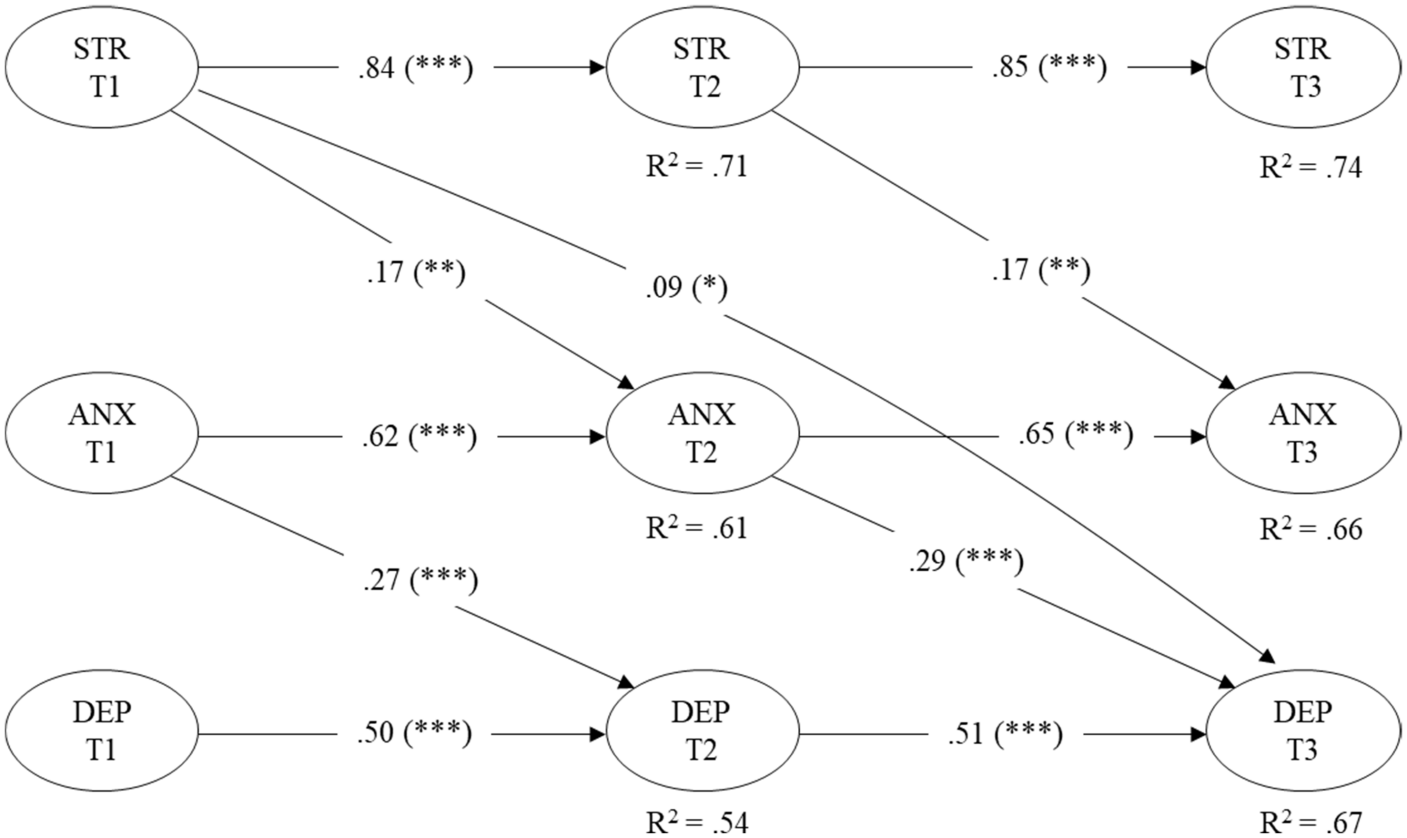
Longitudinal mediation model of anxiety, perceived stress and depression, controlling for fear of COVID-19. All variables are latent constructs. STR, perceived stress; ANX, anxiety; DEP, depression. Time 1 (T1), Time 2 (T2), and Time 3 (T3). Solid lines represent statistically significant paths. Covariances among latent variables at each wave and factor loadings of the latent constructs were omitted for simplicity; all factor loadings ranged between 0.75 and 0.95 (*p* < 0.001). Model fit indices: χ^2^(450) = 870.19, *p* < 0.001, CFI = 0.979, TLI = 0.975, RMSEA = 0.034, 90% CI [0.031, 0.037], SRMR = 0.043. Significance levels: ***p < 0.001, **p < 0.01, *p < 0.05.

Then, following Cole et al. (2005), we constrained all autoregressive and cross-lagged paths to be time-invariant. This decision was based on the premise that, by keeping the time intervals between measurements constant (2 months), there is insufficient theoretical justification to expect variations in the magnitude of the coefficients over time. This simplification enabled us to model the effects between T1 and T2, as well as between T2 and T3, using a single parameter for each trajectory, which favors model parsimony and improves statistical power. To validate this assumption, we compared constrained and unconstrained models using Chi-square tests (Kline, 2016). The resulting loading-path model showed a good fit to the data, *χ^2^*(455) = 891.38, *p* < 0.001, CFI = 0.978, TLI = 0.975, RMSEA = 0.035, 90% CI [0.031, 0.038], SRMR = 0.045. This model was compared with the loading-only model, whose fit was *χ^2^*(450) = 870.19, *p* < 0.001, CFI = 0.979, TLI = 0.975, RMSEA = 0.034, 90% CI [0.031, 0.037], SRMR = 0.043. To evaluate the imposed restrictions, a chi-square difference test was performed between the loading model (χ^2^ = 870.19, df = 450) and the loading-path model (χ^2^ = 891.38, df = 455). The result was *Δχ^2^*(5) = 21.19, with a *p-*value ≈ 0.0011. Although this difference was statistically significant, it should be interpreted with caution, given the sample size (*n* = 805) and the stability of the overall fit indices (ΔCFI = 0.001). This suggests that the constraints do not substantially deteriorate the fit, supporting the choice of the more parsimonious constrained model. The structural paths of the mediation model are illustrated in Figure 2 and summarized in Table 4. As for the autoregressive effects, and given that time invariance was assumed, only the estimates corresponding to the interval T1 to T2 are reported in the text. These were statistically significant for the three latent variables: perceived stress (*β* = 0.84, 95% CI [0.80, 0.87], *p* < 0.001), anxiety (*β* = 0.62, 95% CI [0.50, 0.73], p < 0.001), and depression (*β* = 0.50, 95% CI [0.37, 0.62], *p* < 0.001), indicating moderate-to-high temporal stability. As for the cross-lagged effects, and consistent with the assumption of time invariance, only the estimates corresponding to the interval T1 to T2 are reported. Stress at T1 significantly predicted higher levels of anxiety at T2 (*β* = 0.17, 95% CI [0.06, 0.28], *p* = 0.002), while anxiety at T1 predicted subsequent increases in depression at T2 (*β* = 0.27, 95% CI [0.17, 0.38], p < 0.001). Factor loadings ranged from 0.75 to 0.95 (all with *p* < 0.001), and *R^2^* values ranged from 0.54 to 0.74 (all with *p* < 0.001). Taken together, these findings provide empirical support for H1, as stress at T1 significantly predicted anxiety at T2, and for H2, given that anxiety at T2 was a robust predictor of depression at T3. H3 was also supported, as stress at T1 directly predicted depressive symptoms at T3.

**Table 4:** Standardized path coefficients in the longitudinal mediation model linking stress, anxiety, and depression.

Pathway	*β*	95% CI	*p*-value
Autoregressive effects			
Stress T1 → Stress T2	0.84	[0.80, 0.87]	< 0.001
Stress T2 → Stress T3	0.85	[0.81, 0.89]	< 0.001
Anxiety T1 → Anxiety T2	0.62	[0.50, 0.73]	< 0.001
Anxiety T2 → Anxiety T3	0.65	[0.52, 0.76]	< 0.001
Depression T1 → Depression T2	0.50	[0.37, 0.62]	< 0.001
Depression T2 → Depression T3	0.51	[0.38, 0.63]	< 0.001
Cross-lagged effects			
Stress T1 → Anxiety T2	0.17	[0.06, 0.28]	0.002
Stress T2 → Anxiety T3	0.17	[0.06, 0.29]	0.002
Anxiety T1 → Depression T2	0.27	[0.17, 0.38]	< 0.001
Anxiety T2 → Depression T3	0.29	[0.17, 0.39]	< 0.001
Stress T1 → Depression T3	0.09	[0.01, 0.17]	0.031
Mediation (effects of stress T1 on depression T3)			
Total	0.14	[0.04, 0.22]	0.002
Total Indirect	0.05	[0.02, 0.09]	0.009
Specific Indirect (Stress T1 → Anxiety T2 → Depression T3)	0.05	[0.02, 0.09]	0.009
Direct (Stress T1 → Depression T3)	0.09	[0.01, 0.17]	0.031

Standardized coefficients (*β*) are reported with 95% confidence intervals for autoregressive and cross-lagged effects. For indirect and total effects, 95% bootstrap confidence intervals (CIs) were calculated based on 1,000 iterations. T1 = Time 1, T2 = Time 2, T3 = Time 3. ***p < 0.001; **p < 0.01; *p < 0.05.

### 7.4 Mediation analysis

**Figure 3:**
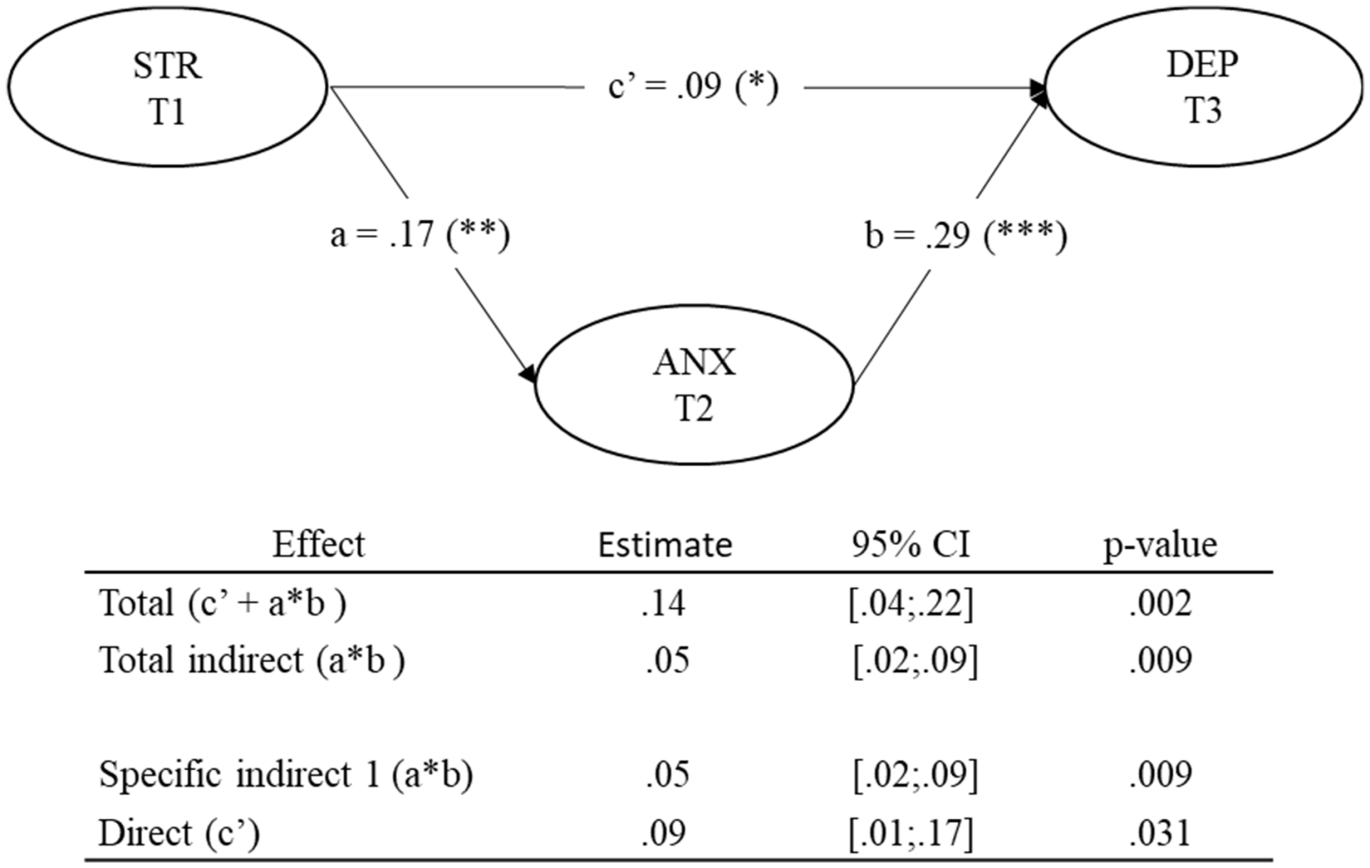
Longitudinal mediation model linking perceived stress (T1) to depression (T3) through anxiety (T2). All variables are latent constructs. STR, perceived stress; ANX, anxiety; DEP, depression. T1, Time 1; T2, Time 2; T3, Time 3. Coefficients are standardized. Solid lines represent statistically significant paths. Indirect and total effects were estimated in Mplus with 95% bootstrap confidence intervals (CIs) based on 1,000 iterations. ***p < 0.001; **p < 0.01; *p < 0.05.

The results also support the central hypothesis of the study, which postulates that anxiety acts as a mediating variable in the longitudinal relationship between perceived stress and depression (see Figure 3 and Table 4). To assess this mediation, a longitudinal model with three temporal measurements was specified, in which perceived stress at T1 predicts anxiety at T2 (trajectory a), in turn, predicts depression symptoms at T3 (trajectory b). We also included the direct trajectory of stress at T1 on depression at T3 (trajectory c’), which allowed us to distinguish between full and partial mediation. Perceived stress at T1 significantly predicted anxiety at T2, *β* = 0.17, 95% CI [0.062, 0.282], *p* = 0.002, and anxiety at T2 significantly predicted depression at T3, *β* = 0.29, 95% CI [0.172, 0.393], *p* < 0.001. The direct effect of perceived stress on depression was also significant, although of smaller magnitude, *β* = 0.08, 95% CI [0.009, 0.167], *p* = 0.031, indicating that the mediation is partial rather than full. The indirect effect of stress on depression, mediated by anxiety, was significant, with a bias-corrected 95% bootstrap confidence interval (1,000 iterations): indirect effect = 0.049, 95% CI [0.016, 0.091], *p* = 0.009. These findings empirically support the hypothesis that perceived stress exerts both direct and indirect effects on subsequent mental health, and that anxiety plays a key role as an emotional mechanism in this process.

To further evaluate the robustness of these findings, we examined whether the final sample size (*n* = 479 completers) provided sufficient statistical power to detect the hypothesized indirect effect. We conducted a post-hoc Monte Carlo simulation with 200,000 replications based on the standardized path estimates from the fitted model (a = 0.172, SE = 0.055; b = 0.286, SE = 0.057). Simulations were implemented in Python using the NumPy library to generate random draws from normal distributions for each path, compute the product a × b, and evaluate its sampling distribution. The proportion of simulated indirect effects with two-tailed *p* < 0.05 provided the estimated power, which was 0.86, exceeding the conventional 0.80 threshold (Selig and Preacher, 2009). This analysis supports the adequacy of the sample size and reinforces the validity of the mediation results. Accordingly, H4 was supported, as anxiety at T2 significantly mediated the longitudinal association between stress at T1 and depression at T3.

## 8 Discussion

Several studies have shown that chronic stress constitutes a robust antecedent of psychological distress, particularly when it is internalized through dysfunctional patterns of emotional and cognitive processing. Consistent with this line of research, our results reveal that anxiety acts as a significant mediating mechanism in the relationship between perceived stress and depression. Specifically, the longitudinal structural model confirmed that higher levels of perceived stress at T1 predict increases in anxiety at T2, which in turn predict higher levels of depression at T3. This finding provides empirical evidence that clarifies a functional sequence proposed theoretically by Beck (1976), Beck et al. (2024) and supported by recent work (Anyan et al., 2017, 2020). However, this sequence has been insufficiently validated using longitudinal designs to date.

### 8.1 Theoretical implications

One of the main theoretical contributions of this study is the empirical validation of a functional sequence originally suggested by Beck’s cognitive model (Beck, 1976; Beck et al., 2024) in which anxiety operates as a sequential mediator linking stress to depression. Previous studies have provided support for this pathway (Anyan et al., 2017, 2018, 2020). However, their reliance on two-wave designs limited the ability to establish temporal precedence or to disentangle intraindividual stability from change. By employing a three-wave longitudinal approach with CLPM models, the present study addresses this gap. It provides more robust evidence for the mediational role of anxiety in non-clinical adult populations. In this framework, stress activates dysfunctional schemas that elicit anxious responses, which, if sustained, evolve into depressive symptomatology. Although this progression was clinically acknowledged in earlier versions of the cognitive model, the role of anxiety as a formal mediating mechanism had not been empirically validated with sufficient rigor. In terms of magnitude, the effects observed in this study were moderate. The path from stress to anxiety (*β* = 0.17) and from anxiety to depression (*β* = 0.29) are comparable to those reported in community and student samples, such as Anyan et al., 2017, *β* = 0.19 and *β* = 0.25) and Jacobson and Newman (2017, *β* = 0.15–0.23 and *β* = 0.18–0.24). By contrast, Kok et al. (2016) reported substantially stronger effects when examining the mediating role of trait anxiety in cardiac surgery patients (*β* = 0.34 and *β* = 0.74), with full mediation of the stress–depression link. These differences can be explained both by the clinical and trauma-exposed nature of their sample and by the use of trait anxiety, a stable personality factor strongly associated with depression, rather than anxiety assessed at a specific time point as in the present study. This comparison underscores the contribution of our work: it demonstrates that Beck’s functional sequence also holds in non-clinical adults under conditions of sustained stress, albeit with more moderate effect sizes, thereby supporting its robustness and generalizability beyond clinical contexts.

First, methodologically, our work strengthens the internal validity of this mediational sequence. Using CLPM modeling, we were able to establish temporal ordering while controlling for intraindividual stability, meeting the classic criteria of mediation (Baron and Kenny, 1986). This refinement not only confirms anxiety’s role within a cognitive–affective trajectory but also represents a concrete contribution to the field by advancing longitudinal methods suited to testing cognitive–emotional processes.

Second, this work contributes to the refinement of the classical cognitive model by clarifying the specific role of anxiety within the stress-depression sequence. Whereas Beck’s original model emphasizes the activation of dysfunctional schemas as an antecedent of depressive states, our findings show that anxiety is not merely a co-occurring symptom, but a mediating mechanism that cognitively transforms the perception of threat into depressive symptomatology. This refinement allows us to describe the model as a temporal sequence with distinct affective phases.

Third, the study contributes to the literature on temporal affective mediation by providing evidence that anxiety acts as a transient emotional state with a prospective effect, which has implications for theories of emotional regulation and the maintenance of negative affect. Thus, it opens the possibility of incorporating this mediation into broader frameworks that integrate dimensions of time, intensity, and emotional reactivity, moving towards a more dynamic understanding of the psychopathological course.

At the same time, it is important to situate these findings within broader theoretical debates. While Beck’s (1976), Beck et al., (1985b) cognitive model identifies maladaptive schemas as a shared substrate for anxiety and depression, it does not specify a temporal sequence. Subsequent frameworks have emphasized co-occurrence rather than sequential ordering. For example, the tripartite model (Clark and Watson, 1991) links anxiety and depression through high negative affect, differing mainly in physiological hyperarousal or low positive affect. Transdiagnostic approaches (Brown et al., 1998) similarly conceptualize them as concurrent expressions of shared vulnerabilities, and epidemiological studies (Kendler et al., 2007) report strong bidirectional associations without temporal ordering. Our findings challenge these perspectives by providing temporal evidence that anxiety prospectively mediates the stress–depression pathway, thereby imposing sequential structure on a relationship often treated as simultaneous or reciprocal.

Unlike the tripartite model (Clark and Watson, 1991), which conceptualizes anxiety and depression as concurrent outcomes of heightened negative affect differentiated only by physiological arousal or diminished positive affect, our findings demonstrate temporal precedence, thereby challenging the assumption of synchronicity and highlighting anxiety’s prospective role in shaping depressive outcomes. Similarly, transdiagnostic approaches (Brown et al., 1998) and epidemiological studies (Kendler et al., 2007) emphasize strong bidirectional associations without specifying temporal ordering; however, the present evidence suggests that stress-related anxiety exerts a directional effect on subsequent depression rather than emerging simultaneously. Beyond these models, rumination theory (Nolen-Hoeksema et al., 2008) and metacognitive frameworks (Wells, 2009) propose cognitive mechanisms, repetitive negative thinking, and dysfunctional metacognitive beliefs that may interact with anxiety in shaping vulnerability to depression. Importantly, empirical findings support this proposition. For instance, Hong (2007) demonstrated that worry (anxious repetitive thought) predicted subsequent increases in both anxiety and depression, whereas rumination showed a more specific association with depressive symptoms. Similarly, Dickson et al. (2012) reported that perseverative worry exacerbates negative affect and facilitates the transition from anxious arousal to depressive mood. These findings illustrate how stress-induced anxiety may trigger worry processes, which in turn reinforce rumination and deepen depressive trajectories. Integrating such evidence strengthens the interpretation that anxiety not only functions as an independent mediator but may also operate sequentially with cognitive vulnerabilities, thereby extending Beck’s framework toward a more dynamic model of risk.

Although our study did not directly test these mechanisms, future research should clarify whether anxiety interacts with repetitive thought processes in parallel or as part of a broader sequential trajectory within Beck’s framework.

Ultimately, a key strength of this study lies in its three-wave longitudinal design, which allows for the evaluation of temporal ordering and indirect effects with greater rigor than cross-sectional or two-wave approaches (Cole and Maxwell, 2003; Little, 2013).

### 8.2 Practical implications

Notably, the study was conducted during the COVID-19 pandemic, a period characterized by unprecedented health, social, and economic stressors. Although our design does not allow us to compare stress levels before and during the pandemic, this context nonetheless provides a meaningful backdrop for interpreting the mediational sequence. Specifically, sustained stressors present in crisis contexts may intensify the transition from anxiety to depression. Framing the findings within this context underscores their real-world significance while recognizing that the results should be interpreted as evidence of the stress, anxiety, and depression pathway under pandemic conditions. Importantly, fear of COVID-19 was explicitly modeled as an exogenous predictor of stress, anxiety, and depression at each wave, ensuring that the reported associations reflect genuine psychological mechanisms rather than pandemic-driven confounding.

From an applied perspective, this study offers relevant implications for clinical settings, community strategies, and the design of public mental health policies. Our findings suggest that anxiety not only coexists with depression but also functions as an early indicator with potential mediating properties. Therefore, preventive strategies should prioritize early detection and clinical management of subclinical anxiety to interrupt its progression to more severe depressive conditions. This orientation aligns with evidence from randomized controlled trials showing that early anxiety symptoms in at-risk individuals can predict the later onset of depressive episodes. This orientation is consistent with the evidence provided by controlled trials that have demonstrated the efficacy of early interventions in at-risk populations (Garber et al., 2009).

In this framework, it is crucial to implement psychoeducational and therapeutic interventions adapted to the social and emotional context in which stress manifests. Cognitive-behavioral programs that consider situational factors have shown promising results, especially when they incorporate training in emotional self-regulation, symptom monitoring, or the provision of containment spaces in clinical and community settings. Nevertheless, implementing such strategies may face significant barriers. In the Chilean context, inequities in access to specialized services, disparities between urban and peri-urban areas, and sociocultural norms that influence help-seeking behaviors represent challenges that limit the scalability and effectiveness of interventions. Acknowledging these barriers highlights the importance of tailoring preventive strategies to the sociocultural and geographical characteristics of the populations they are directed towards, thereby ensuring ecological validity and enhancing their real-world applicability. Specifically, interventions that target anxiety in its early phases may function as critical “disruption points” in the sequential pathway, reducing the likelihood that stress exposure crystallizes into depressive symptomatology.

In addition, the results present an opportunity for the development of public policies aimed at preventing affective disorders from a population-based approach. Educational institutions, primary health services, and employers could implement structured screening programs that identify risk profiles and activate early referral mechanisms to support individuals at risk. Screening programs that systematically detect subclinical anxiety could serve as a feasible, cost-effective strategy to intervene at the sequential stage before depression emerges. In this regard, our findings highlight the value of community- and workplace-based programs that strengthen resilience and stress-management skills in emotionally demanding contexts, as a concrete preventive strategy directly aligned with the mediating role of anxiety identified in this study. In parallel, the development of digital platforms and mobile applications capable of monitoring distress patterns and delivering automated interventions could substantially broaden the preventive reach of traditional clinical strategies. Such tools are particularly valuable for underserved populations or individuals facing barriers to timely access, as they can provide scalable, low-cost, and context-sensitive support that complements face-to-face interventions.

### 8.3 Limitations and future lines of research

The longitudinal design employed in this study represents a significant methodological advance, as it enables the evaluation of temporal relationships between variables, the estimation of prospective mediations, and the control of intraindividual stability. However, this type of design does not allow for the modeling of specific aspects of affective change, such as nonlinear trajectories, cumulative effects, or feedback between constructs. In this sense, latent growth models can be a complementary tool for more accurately capturing intraindividual variability and exploring how mediating effects change over time (Curran et al., 2010; McArdle, 2009).

An additional limitation is that we did not account for potential confounding variables such as stressful life events, clinical comorbidities, or personality traits, which may influence the longitudinal trajectories. Future research could incorporate additional measures or high-resolution methodologies, such as ecological momentary assessment, to better capture these influences.

Also, while this study employed a structural equation model with lagged trajectories (CLPM), which allows for estimating prospective relationships while controlling for intra-individual stability effects, there are more sophisticated models such as the Random Intercept Cross-Lagged Panel Model (RI-CLPM), which allows for explicitly differentiating between intra- and inter-individual processes in longitudinal analysis (Hamaker et al., 2015; Orth et al., 2021). However, the reliable implementation of the RI-CLPM requires at least four measurements to ensure parameter identification and stability, as well as adequate statistical power, conditions that exceed the three-wave design used in this study. In this sense, the choice of the CLPM responds to reasonable methodological criteria that are theoretically consistent with the proposed objectives. Future research with denser time series could benefit from the use of the RI-CLPM to model more accurately the affective dynamics involved in the transition from stress to depression, with anxiety as a sequential mediator.

Although our study was not initially designed with *a priori* power calculation, a post-hoc Monte Carlo analysis (200,000 replications) based on the observed path estimates indicated a statistical power of 0.86 for detecting the hypothesized indirect effect, suggesting that the sample size was adequate for the mediation model.

Beyond methodological considerations, the sociocultural context of data collection constitutes an additional limitation. Cultural characteristics such as prevailing social values, interpersonal relationship patterns, and broader economic conditions may shape how stress, anxiety, and depression unfold and interact. In this study, the sample consisted exclusively of adults residing in the Metropolitan Region of Chile, which restricts the generalizability of the findings to other regions or cultural contexts. This geographic concentration reflects characteristics specific to Santiago, such as higher urbanization, socioeconomic inequality, and the impact of the COVID-19 pandemic. Chile also exhibits a low individualism score, reflecting a more collectivist orientation that may influence coping styles, emotional expression, and the interplay among stress, anxiety, and depression. These contextual features could partly explain variations in the strength or form of the observed pathways. Future research should therefore incorporate more diverse samples across regions, socioeconomic groups, and demographics, as well as cross-cultural comparisons to test whether the mediational sequence identified here generalizes to societies with different cultural orientations and socioeconomic conditions. Additionally, it should examine potential moderators, such as gender, predominant stressors, or educational level. Another relevant limitation relates to the exclusive use of self-reported measures. Although validated instruments were used and possible biases were controlled, this approach may still be susceptible to common method errors and social desirability bias (Podsakoff et al., 2003). Future research should therefore incorporate additional sources of information, with particular emphasis on structured clinical interviews, which provide a more objective diagnostic assessment. Complementary sources, such as third-party reports or physiological indicators, would further strengthen the convergent validity of the findings.

The model analyzed focused exclusively on anxiety as a mediator of the relationship between stress and depressive symptoms, based on a hypothesis derived from Beck’s cognitive model. This decision is informed by methodological and theoretical criteria that favor the use of parsimonious and clearly defined models when analyzing longitudinal data with three waves (Hayes, 2013). Nevertheless, we acknowledge that other psychological processes—such as rumination, attributional styles, and self-efficacy—constitute plausible alternative pathways in the progression from stress to depression. These mechanisms were not included in the present model due to design restrictions; however, their examination through multivariate analyses and longitudinal designs with greater temporal density represents a crucial direction for future research.

Although the present study is explicitly grounded in Beck’s cognitive model, its findings suggest the possibility of exploring complementary conceptual frameworks in future research. In particular, approaches such as rumination theory (Nolen-Hoeksema et al., 2008) and dysfunctional metacognition (Wells, 2009) offer relevant theoretical hypotheses for understanding how anticipatory anxiety might amplify or maintain dysfunctional affective trajectories. However, since these dimensions were neither operationalized nor included in the analytical model proposed here, their mention is limited to an orienting theoretical opening, without constituting a conceptual integration within the present work.

Thus, although the proposed model captures a plausible mediational route within the cognitive framework, it does not pretend to exhaust the multiple possible pathways in the affective progression from stress to depression. Its value lies in offering a parsimonious and empirically tested theoretical sequence, which can serve as a starting point for more comprehensive and comparative models in future research. Finally, although the results obtained align with Beck’s cognitive model, they also open up the possibility of examining similar affective trajectories in other clinical conditions. This projection suggests an exploratory direction towards more integrative models, in which anxiety could play an articulating role within complex emotional sequences. In particular, the hypothesis of a shared matrix of cognitive and emotional vulnerability, proposed by transdiagnostic models such as those of Ingram and Luxton (2005) or the rumination theory of Nolen-Hoeksema et al. (2008), constitutes a promising avenue for future research. However, its empirical validation would require more extensive and multivariate designs, which are beyond the scope of the present study.

Additionally, although attrition analyses suggested no systematic bias in stress, anxiety, depression, or fear of COVID-19, the fact that participants who remained were on average older indicates that the generalizability of our findings may be more limited for younger adults. While this age-related attrition does not compromise the internal validity of the mediation model, it highlights the need for caution when extrapolating results to younger populations.

## 9 Conclusion

The findings demonstrate that anxiety plays a mediating role in the temporal sequence connecting perceived stress with the onset of depressive symptoms. Using a three-wave longitudinal design and a structural model with lagged paths, we identified an emotional sequence in which stress precedes increases in anxiety, which, in turn, predicts higher levels of depressive symptoms. These findings update the classical cognitive model of depression by positioning anxiety as a sequential emotional component that links threat perception with the development of depressive states. This perspective offers a more dynamic understanding of affective disorders by integrating theories of emotion regulation and cognitive vulnerability. In applied terms, the results support the need for preventive strategies focused on subclinical anxiety. Early detection and treatment of these symptoms could interrupt their progression into fully developed depressive conditions. It also highlights the potential for developing clinical, community, or digital tools to monitor and manage these emotional trajectories. Overall, the results contribute to a more accurate understanding of affective trajectories by integrating cognitive and emotional components in a sequential model with potential applications in preventive interventions. In sum, the study offers both a theoretical and practical contribution by proposing a longitudinal mediational model that improves the understanding of psychopathological trajectories by clarifying how stress evolves into depression via anxiety, thus consolidating an affective sequence of high clinical and preventive value.

## Data Availability

The raw data supporting the conclusions of this article will be made available by the authors, without undue reservation.

## Author’s note

This work is part of Gustavo Marcos Escobar’s doctoral research conducted within the Ph.D. program in Management at Business School, Universidad Adolfo Ibáñez.
