# Supplementary material for: Understanding the transition from stress to depression: a longitudinal mediational analysis of anxiety in adults from the Metropolitan Region of Chile
Source: Front Psychol. 2025 Sep 26;16:1668518. doi: 10.3389/fpsyg.2025.1668518 (PMC12511094; doi:10.3389/fpsyg.2025.1668518)
Supplement: Supplementary file 1 [file Data_Sheet_1.pdf]

Supplementary Material – Use of Generative AI

Initial Prompt (Language Editing Only)

Prompt used at the beginning of the editing process (stylistic revision only):

Act as an expert academic editor specialized in English-language scientific writing in psychology and affective neuroscience. Your task is to review a manuscript written entirely by human authors. You must not invent content, modify theoretical or empirical sections, or introduce new ideas. Your role is strictly limited to improving grammar, punctuation, fluency, and academic tone.

When suggesting edits, only modify individual words or short expressions (not entire sentences). Use the following format:

– Words to be removed: marked with a ~~strikethrough~~

– Words to be improved or added: marked in bold

Do not apply automatic or structural edits. Human authors will handle coherence, flow, and final integration manually.

Final Prompt (Citation–Reference Consistency Check)

Prompt used at the end of the process to verify citation–reference consistency:

Act as an editorial assistant with expertise in APA 7th edition. Your task is to confirm that all in-text citations have a corresponding entry in the reference list, and that all references listed are cited in the manuscript. Do not invent or suggest new sources.

Identify the following:

– In-text citations missing from the reference list

– Reference list entries not cited in the manuscript

Do not make content changes. The authors will manually review and resolve any inconsistencies that arise.
